# Impact of the COVID-19 Pandemic on Emergency Department Visits — United States, January 1, 2019–May 30, 2020

**DOI:** 10.15585/mmwr.mm6923e1

**Published:** 2020-06-12

**Authors:** Kathleen P. Hartnett, Aaron Kite-Powell, Jourdan DeVies, Michael A. Coletta, Tegan K. Boehmer, Jennifer Adjemian, Adi V. Gundlapalli, National Syndromic Surveillance Program Community of Practice

**Affiliations:** ^1^Innovation, Technology, and Analytics Task Force, CDC COVID-19 Response Team; ^2^Division of Health Informatics and Surveillance, Center for Surveillance, Epidemiology, and Laboratory Services, CDC; ^3^Division of Environmental Health Science and Practice, National Center for Environmental Health, CDC; ^4^Public Health Informatics Office, Center for Surveillance, Epidemiology, and Laboratory Services, CDC.

On March 13, 2020, the United States declared a national emergency to combat coronavirus disease 2019 (COVID-19). As the number of persons hospitalized with COVID-19 increased, early reports from Austria (*1*), Hong Kong (*2*), Italy (*3*), and California (*4*) suggested sharp drops in the numbers of persons seeking emergency medical care for other reasons. To quantify the effect of COVID-19 on U.S. emergency department (ED) visits, CDC compared the volume of ED visits during four weeks early in the pandemic March 29–April 25, 2020 (weeks 14 to 17; the early pandemic period) to that during March 31–April 27, 2019 (the comparison period). During the early pandemic period, the total number of U.S. ED visits was 42% lower than during the same period a year earlier, with the largest declines in visits in persons aged ≤14 years, females, and the Northeast region. Health messages that reinforce the importance of immediately seeking care for symptoms of serious conditions, such as myocardial infarction, are needed. To minimize SARS-CoV-2, the virus that causes COVID-19, transmission risk and address public concerns about visiting the ED during the pandemic, CDC recommends continued use of virtual visits and triage help lines and adherence to CDC infection control guidance.

To assess trends in ED visits during the pandemic, CDC analyzed data from the National Syndromic Surveillance Program (NSSP), a collaborative network developed and maintained by CDC, state and local health departments, and academic and private sector health partners to collect electronic health data in real time. The national data in NSSP includes ED visits from a subset of hospitals in 47 states (all but Hawaii, South Dakota, and Wyoming), capturing approximately 73% of ED visits in the United States able to be analyzed at the national level. During the most recent week, 3,552 EDs reported data. Total ED visit volume, as well as patient age, sex, region, and reason for visit were analyzed.

Weekly number of ED visits were examined during January 1, 2019–May 30, 2020. In addition, ED visits during two 4-week periods were compared using mean differences and ratios. The change in mean visits per week during the early pandemic period and the comparison period was calculated as the mean difference in total visits in a diagnostic category between the two periods, divided by 4 weeks ([visits in diagnostic category {early pandemic period} – visits in diagnostic category {comparison period}]/4). The visit prevalence ratio (PR) was calculated for each diagnostic category as the proportion of ED visits during the early pandemic period divided by the proportion of visits during the comparison period ([visits in category {early pandemic period}/all visits {early pandemic period}]/[visits in category {comparison period}/all visits {comparison period}]). All analyses were conducted using R software (version 3.6.0; R Foundation).

Reason for visit was analyzed using a subset of records that had at least one specific, billable *International Classification of Diseases, Tenth Revision, Clinical Modification* (ICD-10-CM) code. In addition to Hawaii, South Dakota, and Wyoming, four states (Florida, Louisiana, New York outside New York City, and Oklahoma), two California counties reporting to the NSSP (Santa Cruz and Solano), and the District of Columbia were also excluded from the diagnostic code analysis because they did not report diagnostic codes during both periods or had differences in completeness of codes between 2019 and 2020. Among eligible visits for the diagnostic code analysis, 20.3% without a valid ICD-10-CM code were excluded. ED visits were categorized using the Clinical Classifications Software Refined tool (version 2020.2; Healthcare Cost and Utilization Project), which combines ICD-10-CM codes into clinically meaningful groups (*5*). A visit with multiple ICD-10-CM codes could be included in multiple categories; for example, a visit by a patient with diabetes and hypertension would be included in the category for diabetes and the category for hypertension. Because COVID-19 is not yet classified in this tool, a custom category, defined as any visit with the ICD-10-CM code for confirmed COVID-19 diagnosis (U07.1), was created (*6*). The analysis was limited to the top 200 diagnostic categories during each period.

The lowest number of visits reported to NSSP occurred during April 12–18, 2020 (week 16). Although visits have increased since the nadir, the most recent complete week (May 24–30, week 22) remained 26% below the corresponding week in 2019 (Figure 1). The number of ED visits decreased 42%, from a mean of 2,099,734 per week during March 31–April 27, 2019, to a mean of 1,220,211 per week during the early pandemic period of March 29–April 25, 2020. Visits declined for every age group (Figure 2), with the largest proportional declines in visits by children aged ≤10 years (72%) and 11–14 years (71%). Declines in ED visits varied by U.S. Department of Health and Human Services region,* with the largest declines in the Northeast (Region 1, 49%) and in the region that includes New Jersey and New York (Region 2, 48%) (Figure 2). Visits declined 37% among males and 45% among females across all NSSP EDs between the comparison and early pandemic periods.

**FIGURE 1:**
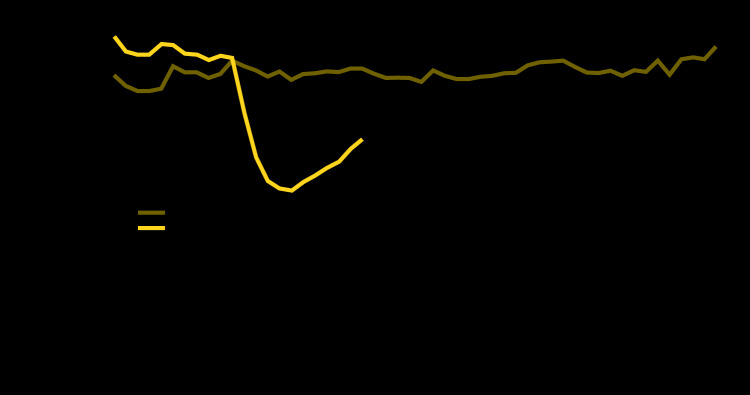
Weekly number of emergency department (ED) visits — National Syndromic Surveillance Program, United States,* January 1, 2019– May 30, 2020^†^ * Hawaii, South Dakota, and Wyoming are not included. ^†^ Vertical lines indicate the beginning and end of the 4-week coronavirus disease 2019 (COVID-19) early pandemic period (March 29–April 25, 2020) and the comparison period (March 31–April 27, 2019).

**FIGURE 2:**
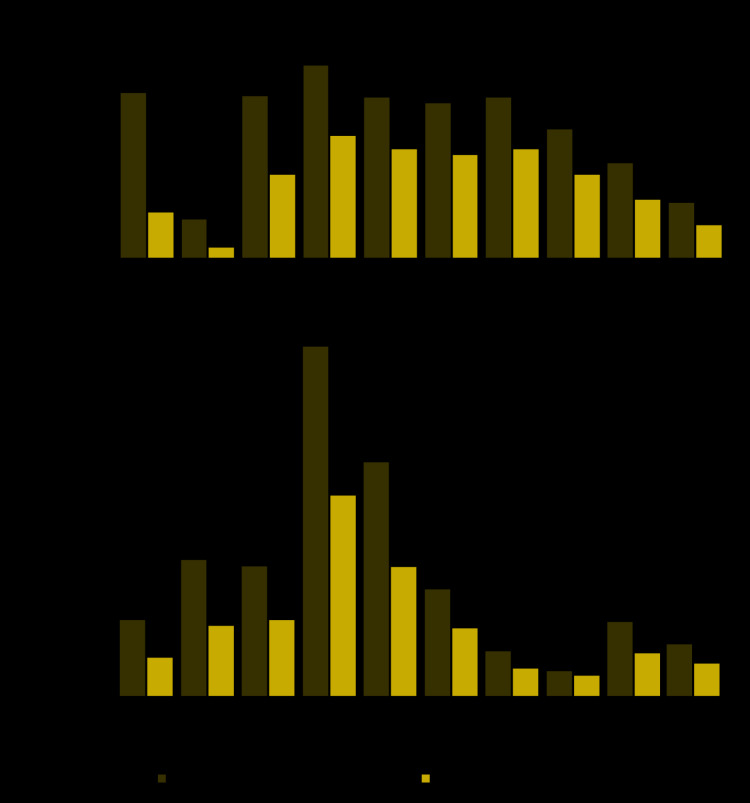
Emergency department (ED) visits, by age group (A) and U.S. Department of Health and Human Services (HHS) region* (B) — National Syndromic Surveillance Program, United States,^†^ March 31–April 27, 2019 (comparison period) and March 29–April 25, 2020 (early pandemic period) * *Region 1*: Connecticut, Maine, Massachusetts, New Hampshire, Rhode Island, and Vermont; *Region 2*: New Jersey and New York; *Region 3*: Delaware, District of Columbia, Maryland, Pennsylvania, Virginia, and West Virginia; *Region 4*: Alabama, Florida, Georgia, Kentucky, Mississippi, North Carolina, South Carolina, and Tennessee; *Region 5*: Illinois, Indiana, Michigan, Minnesota, Ohio, and Wisconsin; *Region 6*: Arkansas, Louisiana, New Mexico, Oklahoma, and Texas; *Region 7*: Iowa, Kansas, Missouri, and Nebraska; *Region 8*: Colorado, Montana, North Dakota, and Utah; *Region 9*: Arizona, California, and Nevada; *Region 10*: Alaska, Idaho, Oregon, and Washington. ^†^ Hawaii, South Dakota, and Wyoming are not included.

Among all ages, an increase of >100 mean visits per week from the comparison period to the early pandemic period occurred in eight of the top 200 diagnostic categories (Table). These included 1) exposure, encounters, screening, or contact with infectious disease (mean increase 18,834 visits per week); 2) COVID-19 (17,774); 3) other general signs and symptoms (4,532); 4) pneumonia not caused by tuberculosis (3,911); 5) other specified and unspecified lower respiratory disease (1,506); 6) respiratory failure, insufficiency, or arrest (776); 7) cardiac arrest and ventricular fibrillation (472); and 8) socioeconomic or psychosocial factors (354). The largest declines were in visits for abdominal pain and other digestive or abdomen signs and symptoms (–66,456), musculoskeletal pain excluding low back pain (–52,150), essential hypertension (–45,184), nausea and vomiting (–38,536), other specified upper respiratory infections (–36,189), sprains and strains (–33,709), and superficial injuries (–30,918). Visits for nonspecific chest pain were also among the top 20 diagnostic categories for which visits decreased (–24,258). Although not in the top 20 declining diagnoses, visits for acute myocardial infarction also declined (–1,156).

**TABLE:** Differences in mean weekly numbers of emergency department (ED) visits* for diagnostic categories with the largest increases or decreases^†^ and prevalence ratios^§^ comparing the proportion of ED visits in each diagnostic category, for categories with the highest and lowest ratios — National Syndromic Surveillance Program, United States,^¶^ March 31–April 27, 2019 (comparison period) and March 29–April 25, 2020 (early pandemic period)

Diagnostic category	Change in mean no. of weekly ED visits*	Prevalence ratio (95% CI)^§^
**All categories with higher visit counts during the early pandemic period**		
Exposure, encounters, screening, or contact with infectious disease**	18,834	3.79 (3.76–3.83)
COVID-19	17,774	—
Other general signs and symptoms**	4,532	1.87 (1.86–1.89)
Pneumonia (except that caused by tuberculosis)**	3,911	1.91 (1.90–1.93)
Other specified and unspecified lower respiratory disease**	1,506	1.99 (1.96–2.02)
Respiratory failure, insufficiency, arrest**	776	1.76 (1.74–1.78)
Cardiac arrest and ventricular fibrillation**	472	1.98 (1.93–2.03)
Socioeconomic or psychosocial factors**	354	1.78 (1.75–1.81)
**Other top 10 highest prevalence ratios**		
Mental and substance use disorders, in remission**	6	1.69 (1.64–1.75)
Other specified encounters and counseling**	22	1.69 (1.67–1.72)
Stimulant-related disorders**	−189	1.65 (1.62–1.67)
**Top 20 categories with lower visit counts during the early pandemic period**		
Abdominal pain and other digestive or abdomen signs and symptoms	−66,456	0.93 (0.93–0.93)
Musculoskeletal pain, not low back pain	−52,150	0.81 (0.81–0.82)
Essential hypertension	−45,184	1.11 (1.10–1.11)
Nausea and vomiting	−38,536	0.85 (0.84–0.85)
Other specified upper respiratory infections	−36,189	0.82 (0.81–0.82)
Sprains and strains, initial encounter**^††^**	−33,709	0.61 (0.61–0.62)
Superficial injury; contusion, initial encounter	−30,918	0.85 (0.84–0.85)
Personal or family history of disease	−28,734	1.21 (1.20–1.22)
Headache, including migraine	−27,458	0.85 (0.84–0.85)
Other unspecified injury	−25,974	0.84 (0.83–0.84)
Nonspecific chest pain	−24,258	1.20 (1.20–1.21)
Tobacco-related disorders	−23,657	1.19 (1.18–1.19)
Urinary tract infections	−23,346	1.02 (1.02–1.03)
Asthma	−20,660	0.91 (0.90–0.91)
Disorders of lipid metabolism	−20,145	1.12 (1.11–1.13)
Spondylopathies/Spondyloarthropathy (including infective)	−19,441	0.78 (0.77–0.79)
Otitis media**^††^**	−17,852	0.35 (0.34–0.36)
Diabetes mellitus without complication	−15,893	1.10 (1.10–1.11)
Skin and subcutaneous tissue infections	−15,598	1.01 (1.00–1.02)
Chronic obstructive pulmonary disease and bronchiectasis	−15,520	1.05 (1.04–1.06)
**Other top 10 lowest prevalence ratios**		
Influenza**^††^**	−12,094	0.16 (0.15–0.16)
No immunization or underimmunization**^††^**	−1,895	0.28 (0.27–0.30)
Neoplasm-related encounters**^††^**	−1,926	0.40 (0.39–0.42)
Intestinal infection**^††^**	−5,310	0.52 (0.51–0.54)
Cornea and external disease**^††^**	−9,096	0.54 (0.53–0.55)
Sinusitis**^††^**	−7,283	0.55 (0.54–0.56)
Acute bronchitis**^††^**	−15,470	0.59 (0.58–0.60)
Noninfectious gastroenteritis**^††^**	−11,572	0.63 (0.62–0.64)

**Abbreviations:** CI = confidence interval; COVID-19 = coronavirus disease 2019.

* The change in visits per week during the early pandemic and comparison periods was calculated as the difference in total visits between the two periods, divided by 4 weeks ([visits in diagnostic category, {early pandemic period} – visits in diagnostic category, {comparison period}] / 4).

^†^ Analysis is limited to the 200 most common diagnostic categories. All eight diagnostic categories with an increase of >100 in the mean number of visits nationwide in the early pandemic period are shown. The top 20 categories with decreasing visit counts are shown.

^§^ Ratio calculated as the proportion of all ED visits in each diagnostic category during the early pandemic period, divided by the proportion of all ED visits in that category during the comparison period ([visits in category {early pandemic period}/all visits {early pandemic period})/(visits in category {comparison period}/all visits {comparison period}]). Ratios >1 indicate a higher proportion of visits in that category during the early pandemic period than the comparison period; ratios <1 indicate a lower proportion during the early pandemic than during the comparison period. Analysis is limited to the 200 most common diagnostic categories. The 10 categories with the highest and lowest ratios are shown.

^¶^ Florida, Hawaii, Louisiana, New York outside of New York City, Oklahoma, South Dakota, Wyoming, Santa Cruz and Solano counties in California, and the District of Columbia are not included.

** Top 10 highest prevalence ratios; higher proportion of visits in the early pandemic period than the comparison period.

^††^ Top 10 lowest prevalence ratios; lower proportion of visits in the early pandemic period than the comparison period.

During the early pandemic period, the proportion of ED visits for exposure, encounters, screening, or contact with infectious disease compared with total visits was nearly four times as large as during the comparison period (Table) (prevalence ratio [PR] = 3.79, 95% confidence interval [CI] = 3.76–3.83). The other diagnostic categories with the highest proportions of visits during the early pandemic compared with the comparison period were other specified and unspecified lower respiratory disease, which did not include influenza, pneumonia, asthma, or bronchitis (PR = 1.99; 95% CI = 1.96–2.02), cardiac arrest and ventricular fibrillation (PR = 1.98; 95% CI = 1.93–2.03), and pneumonia not caused by tuberculosis (PR = 1.91; 95% CI = 1.90–1.93). Diagnostic categories that were recorded less commonly during the early pandemic period included influenza (PR = 0.16; 95% CI = 0.15–0.16), no immunization or underimmunization (PR = 0.28; 95% CI = 0.27–0.30), otitis media (PR = 0.35; 95% CI = 0.34–0.36), and neoplasm-related encounters (PR = 0.40; 95% CI = 0.39–0.42).

In the 2019 comparison period, 12% of all ED visits were in children aged ≤10 years old, compared with 6% during the early pandemic period. Among children aged ≤10 years, the largest declines were in visits for influenza (97% decrease), otitis media (85%), other specified upper respiratory conditions (84%), nausea and vomiting (84%), asthma (84%), viral infection (79%), respiratory signs and symptoms (78%), abdominal pain and other digestive or abdomen symptoms (78%), and fever (72%). Mean weekly visits with confirmed COVID-19 diagnoses and screening for infectious disease during the early pandemic period were lower among children than among adults. Among all ages, the diagnostic categories with the largest changes (abdominal pain and other digestive or abdomen signs and symptoms, musculoskeletal pain, and essential hypertension) were the same in males and females, but declines in those categories were larger in females than males. Females also had large declines in visits for urinary tract infections (–19,833 mean weekly visits).

## Discussion

During an early 4-week interval in the COVID-19 pandemic, ED visits were substantially lower than during the same 4-week period during the previous year; these decreases were especially pronounced for children and females and in the Northeast. In addition to diagnoses associated with lower respiratory disease, pneumonia, and difficulty breathing, the number and ratio of visits (early pandemic period versus comparison period) for cardiac arrest and ventricular fibrillation increased. The number of visits for conditions including nonspecific chest pain and acute myocardial infarction decreased, suggesting that some persons could be delaying care for conditions that might result in additional mortality if left untreated. Some declines were in categories including otitis media, superficial injuries, and sprains and strains that can often be managed through primary or urgent care. Future analyses will help clarify the proportion of the decline in ED visits that were not preventable or avoidable such as those for life-threatening conditions, those that were manageable through primary care, and those that represented actual reductions in injuries or illness attributable to changing activity patterns during the pandemic (such as lower risks for occupational and motor vehicle injuries or other infectious diseases).

The striking decline in ED visits nationwide, with the highest declines in regions where the pandemic was most severe in April 2020, suggests that the pandemic has altered the use of the ED by the public. Persons who use the ED as a safety net because they lack access to primary care and telemedicine might be disproportionately affected if they avoid seeking care because of concerns about the infection risk in the ED.

Syndromic surveillance has important strengths, including automated electronic reporting and the ability to track outbreaks in real time (*7*). Among all visits, 74% are reported within 24 hours, with 75% of discharge diagnoses typically added to the record within 1 week.

The findings in this report are subject to at least four limitations. First, hospitals reporting to NSSP change over time as facilities are added, and more rarely, as they close (*8*). An average of 3,173 hospitals reported to NSSP nationally in April 2019, representing an estimated 66% of U.S. ED visits, and an average of 3,467 reported in April 2020, representing 73% of ED visits. Second, diagnostic categories rely on the use of specific codes, which were missing in 20% of visits and might be used inconsistently across hospitals and providers, which could result in misclassification. The COVID-19 diagnosis code was introduced recently (April 1, 2020) and timing of uptake might have differed across hospitals (*6*). Third, NSSP coverage is not uniform across or within all states; in some states nearly all hospitals report, whereas in others, a lower proportion statewide or only those in certain counties report. Finally, because this analysis is limited to ED visit data, the proportion of persons who did not visit EDs but received treatment elsewhere is not captured.

Health care systems should continue to address public concern about exposure to SARS-CoV-2 in the ED through adherence to CDC infection control recommendations, such as immediately screening every person for fever and symptoms of COVID-19, and maintaining separate, well-ventilated triage areas for patients with and without signs and symptoms of COVID-19 (*9*). Wider access is needed to health messages that reinforce the importance of immediately seeking care for serious conditions for which ED visits cannot be avoided, such as symptoms of myocardial infarction. Expanded access to triage telephone lines that help persons rapidly decide whether they need to go to an ED for symptoms of possible COVID-19 infection and other urgent conditions is also needed. For conditions that do not require immediate care or in-person treatment, health care systems should continue to expand the use of virtual visits during the pandemic (*10*).

**Summary**

**What is already known about this topic?**

The National Syndromic Surveillance Program (NSSP) collects electronic health data in real time.

**What is added by this report?**

NSSP found that emergency department (ED) visits declined 42% during the early COVID-19 pandemic, from a mean of 2.1 million per week (March 31–April 27, 2019) to 1.2 million (March 29–April 25, 2020), with the steepest decreases in persons aged ≤14 years, females, and the Northeast. The proportion of infectious disease–related visits was four times higher during the early pandemic period.

**What are the implications for public health practice?**

To minimize SARS-CoV-2 transmission risk and address public concerns about visiting the ED during the pandemic, CDC recommends continued use of virtual visits and triage help lines and adherence to CDC infection control guidance.

## Footnotes

* https://www.hhs.gov/about/agencies/iea/regional-offices/index.html.
